# Effect of Voice and Articulation Parameters of a Home-Based Serious Game for Speech Therapy in Children With Articulation Disorder: Prospective Single-Arm Clinical Trial

**DOI:** 10.2196/49216

**Published:** 2023-10-11

**Authors:** Seong-Yeol Kim, Minji Song, Yunju Jo, Youngjae Jung, Heecheon You, Myoung-Hwan Ko, Gi-Wook Kim

**Affiliations:** 1 Department of Physical Medicine and Rehabilitation Jeonbuk National University Medical School Jeonju-si Republic of Korea; 2 Department of Speech-Language Therapy, Graduate School Jeonbuk National University Jeonju Republic of Korea; 3 Research Institute of Clinical Medicine Biomedical Research Institute of Jeonbuk National University Hospital Jeonju-si Republic of Korea; 4 Department of Industrial and Management Engineering Pohang University of Science and Technology Pohang Republic of Korea

**Keywords:** articulation disorder, home-based therapy, serious game, children, speech, voice

## Abstract

**Background:**

Articulation disorder decreases the clarity of language and causes a decrease in children’s learning and social ability. The demand for non–face-to-face treatment is increasing owing to the limited number of therapists and geographical or economic constraints. Non–face-to-face speech therapy programs using serious games have been proposed as an alternative.

**Objective:**

The aim of this study is to investigate the efficacy of home therapy on logopedic and phoniatric abilities in children with articulation disorder using the Smart Speech game interface.

**Methods:**

This study is a prospective single-arm clinical trial. Children with articulation disorders, whose Urimal Test of Articulation and Phonology (U-TAP) was –2 SDs or less and the Receptive and Expressive Vocabulary Test score was –1 SD or more, were enrolled. A preliminary evaluation (E0) was conducted to check whether the children had articulation disorders, and for the next 4 weeks, they lived their usual lifestyle without other treatments. Prior to the beginning of the training, a pre-evaluation (E1) was performed, and the children trained at home for ≥30 minutes per day, ≥5 times a week, over 4 weeks (a total of 20 sessions). The Smart Speech program comprised oral exercise training, breathing training, and speech training; the difficulty and type of the training were configured differently according to the participants’ articulation error, exercise, and vocal ability. After the training, postevaluation (E2) was performed using the same method. Finally, 8 weeks later, postevaluation (E3) was performed as a follow-up. A voice evaluation included parameters such as maximum phonation time (MPT), fundamental frequency (F_0_), jitter, peak air pressure (relative average perturbation), pitch, intensity, and voice onset time. Articulation parameters included a percentage of correct consonants (PCC; U-TAP word-unit PCC, U-TAP sentence-unit PCC, and three-position articulation test) and alternate motion evaluation (diadochokinesis, DDK). Data obtained during each evaluation (E1-E2-E3) were compared.

**Results:**

A total of 13 children with articulation disorders aged 4-10 years were enrolled in the study. In voice parameters, MPT, jitter, and pitch showed significant changes in repeated-measures ANOVA. However, only MPT showed significant changes during E1-E2 (*P=*.007) and E1-E3 (*P*=.004) in post hoc tests. Other voice parameters did not show significant changes. In articulation parameters, U-TAP, three-position articulation test (TA), and DDK showed significant changes in repeated-measures ANOVA. In post hoc tests, U-TAP (word, sentence) and TA showed significant changes during E1-E2 (*P=*.003, .04, and .01) and E1-E3 (*P*=.001, .03, and .003), and DDK showed significant changes during E1-E2 only (*P*=.03).

**Conclusions:**

Home-based serious games can be considered an alternative treatment method to improve language function.

**Trial Registration:**

Clinical Research Information Service KCT0006448; https://cris.nih.go.kr/cris/search/detailSearch.do/20119

## Introduction

Articulation disorder is a condition characterized by difficulties in producing speech sounds due to a structural abnormality or a neurological or auditory cause. Among children aged 3-11 years, approximately 75% show an articulation problem that demands treatment [1]. Speech and language disorders reduce children’s academic performance and can cause inequality in social and job opportunities in adulthood [2]. Early intervention in children with speech disorders can prevent several problems that may arise later. Effective speech therapy requires treatment sessions to be individualized, frequent, and intensive [3].

The goal of the treatment of articulation disorder is to train the patient to maximize verbal clarity in articulation to ensure an adequate understanding by the listener to increase the efficiency of the function of mutual communication, which would reinforce motivation toward linguistic expressions [4].

Conventionally, treating articulation disorder involves 2 main approaches: the phonetic approach, which focuses on the physiological aspects of the articulation problem, and the phonological approach, in which the treatment focuses on the linguistic aspects of the problem. The phonetic approach includes the phonetic placement method, sensorimotor training, paired-stimuli technique, motor skill learning, and biofeedback technique. The phonological approach to speech therapy includes various methods, such as the phonemic contrast method, cycle training, and Metaphon therapy. The critical point for children with articulation disorders is a suitable early diagnosis and interventions by specialists [5]. Prior to the treatment of articulation disorder, the clinician conducts assessments of voice and articulation to obtain data from the children. The most commonly used parameters for voice evaluation include maximum phonation time (MPT), frequency (F_0_), jitter, relative average perturbation (RAP), pitch, intensity, and voice onset time (VOT). Moreover, commonly used parameters for articulation include percentage of correct consonants (PCC) regarding Urimal Test of Articulation and Phonology (U-TAP) and TA and the diadochokinesis (DDK) rate [6]. In principle, speech therapy is generally performed in face-to-face situations; however, the mode of delivery can vary depending on the situation [7]. Non–face-to-face speech therapy was initially proposed as an alternative method in countries with huge lands, such as the United States and Australia, even before the pandemic. In fact, studies have shown that this method can be effective in treating patients with neuro-lingual disorders, such as dysarthria and apraxia [8].

A computer-assisted home therapy has several advantages. It can offer personalized treatment to each patient, increase accessibility for residents in far-flung regions, and provide intensive training and autonomy, which is impossible when using conventional programs [9]. This could lead to increased treatment effects. A domestic study has found that applying non–face-to-face therapy to children with speech disorders who show articulation errors improved consonant accuracy of the target phoneme [10].

The definition of a functional game varies; nonetheless, it is generally known as a type of game to achieve a goal other than play using the same “motivational effect to cause voluntary participation,” “fun,” and “immersion” as in any game [11]. Smart Speech (Humanopia Co) is a functional game program for vocalization and articulation training designed to assist logopedics. The program allows repetitive and intensive treatments by way of the installed games. Jo et al [12] have treated adult patients with dysarthria using Smart Speech and observed significant positive changes in the following acoustic variables: pitch, intensity, jitter, RAP, and MPT. Additionally, they reported significantly increased scores for word-unit consonant accuracy, articulation accuracy, and sentence-unit consonant accuracy [12]. Currently, several studies have evaluated the efficacy for adults; however, only a few studies have assessed it for children.

This study investigated the efficacy of home therapy on logopedic and phoniatric abilities in children with articulation disorders using the Smart Speech game interface for speech therapy.

## Methods

### Ethical Considerations

This prospective single-arm clinical trial was registered and approved by the institutional review board of Jeonbuk National University Hospital (2019-02-026-015).

### Participants

The study participants included children with articulation disorder who visited our institution’s Department of Rehabilitation Medicine between September 2019 and January 2021. The participants were recruited via an advertisement. The inclusion criteria were as follows: (1) children aged 4 years or older and 12 years or younger; (2) U-TAP score of ≤–2 SD; (3) Receptive and Expressive Vocabulary Test score of ≥–1 SD; and (4) children showing no severe internal pathology without treatment. Before proceeding with the experiment, the participants and their guardians were provided with a full explanation regarding the purpose of the study, procedure, required time, and compensation, and only those who consented to participate were included in the experiment. Since it was a study involving children, signed informed consent was obtained from their guardians.

### Study Protocol

#### Overview

**Figure 1:**
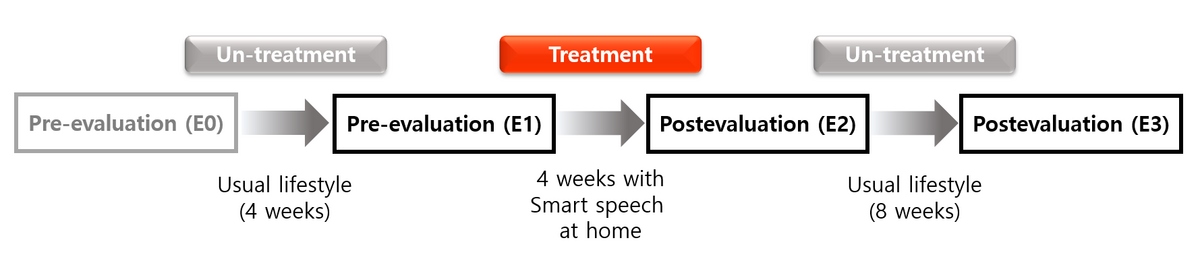
Study protocol.

This study was based on an exploratory design with the following phases: pre-evaluation 1 (E0), pre-evaluation 2 (E1), postevaluation 1 (E2), and postevaluation 2 (E3), in the given order (Figure 1).

#### Pre-Evaluation 1 (E0)

Considering that the study participants were children, we conducted a preliminary evaluation (E0) to adapt the evaluation method before measuring the baseline (E1). The participants’ voice, articulation, and alternate motion were assessed in the E0 phase.

#### Pre-Evaluation 2 (E1)

Before treatment using the Smart Speech program, following phase E0 and 4 weeks of usual lifestyle, the same variables as in E0 were assessed in the pre-evaluation 2 (E1) phase within 3 days.

#### Treatment and Postevaluation 1 (E2)

After the E1 phase and within 1 week, the same participants were provided with a laptop with the Smart Speech program and a microphone and were guided to perform the program at home for ≥30 min per day, ≥5 times a week, for the 4-week period (a total of 20 sessions). In addition, the participants were instructed to record the time of training on the set log during the 4-week period and have telephone counseling with the speech therapist once a week. After the treatment and within 3 days, postevaluation 1 (E2) was performed.

#### Postevaluation 2 (E3)

After E2 and 8 weeks of usual lifestyle, the same variables were assessed in postevaluation 2 (E3) to verify whether the changes induced by the treatment had been retained.

### Intervention Procedures

The Smart Speech is a serious game program for vocalization and articulation training designed to assist logopedics. Structured games relate to oral exercise, breathing, vocalization, and articulation training. The game for vocalization training is subcategorized into sound duration, intensity, pitch, syllable, and word training. Before the intervention, the guardians are given the necessary instruction and training. Each session is designed to provide oral exercises, breathing, vocalization, word, and syllable training in the given order.

### Oral Exercise Training

**Figure 2:**
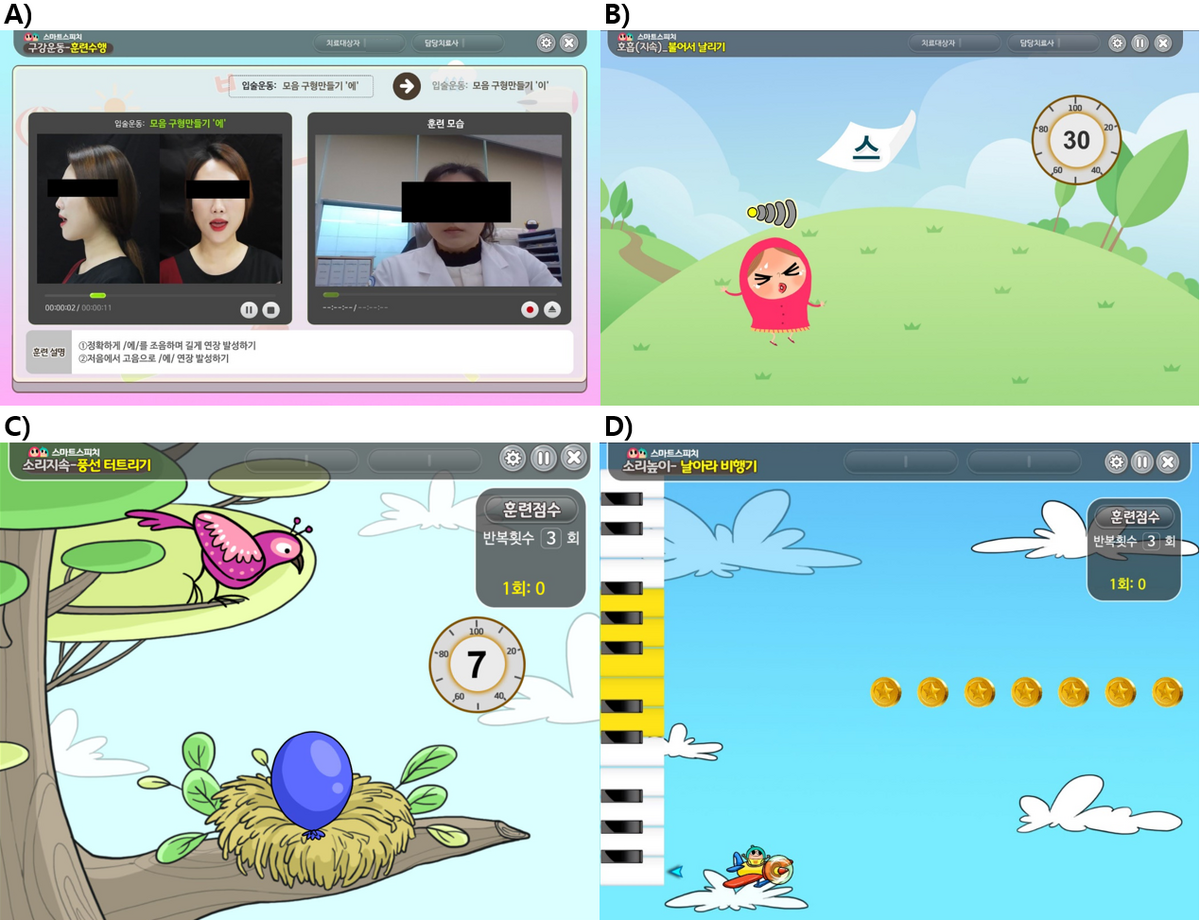
Training program in Smart Speech. Training program in the Smart Speech (Humanopia Co, Korea). (A) In oral exercise training, participants are guided to follow the example on the left screen. They can check their appearance on the right screen and compare the differences with examples. (B) To improve breathing, participants keep breathing for a few seconds continuously so that the paper on the screen does not fall on the floor. (C) In sound duration training, participants can blow up balloons or blow out candles by maintaining a long vocalization in one breath. (D) Participants continued vocalization with the given pitch to fly the birds or airplanes seen on the screen in sound pitch training.

The training consisted of lip, tongue, and chin exercises. In addition, the participants were instructed to watch a recorded video and imitate what they saw while checking how they appeared on the screen. According to the participants’ unique articulation errors and their ability to perform oral exercises, we could select the target of intensive training (Figure 2A).

### Breathing Training

The difficulty level was divided into easy, moderate, and difficult, and the duration could be selected. The training was performed by setting the difficulty level and duration based on the participants’ articulation errors and vocalization ability (Figure 2B).

### Speech Training

#### Duration

Increasing the MPT duration was used to train long vocalization maintenance in a single breath. As the duration and number of repetitions could be set, the difficulty level of the training was adjusted based on the participants’ individual abilities (Figure 2C).

#### Intensity

We could train participants to maintain a suitable voice intensity for a set time by controlling the intensity. As the duration and number of repetitions could be set, the difficulty level of the training was adjusted based on the participants’ individual abilities.

#### Pitch

Controlling the pitch was used to train the ability to maintain a suitable voice pitch for a set time. As the duration and number of repetitions could be set, the difficulty level of the training was adjusted based on the participants’ individual abilities (Figure 2D).

#### Word-Unit Training

In the word-unit training, the user selected a target word to initiate the program and then could set the duration and number of repetitions. The training was designed to allow words containing the target phoneme as the initial, middle, or final consonant to be selected according to the unique articulation errors exhibited by the participants.

#### Syllable Unit Training

In the syllable unit training, the constituent programs allowed the vocalization of a sound with one or more syllables with consonants rather than the simple vocalization of vowels. The programs could be selected according to the phonetic and articulatory abilities of the participants.

### Outcome Measurement

#### Instruments

The voice data were recorded at the Department of Rehabilitation Medicine Language Therapy Laboratory, where external noise was maximally prevented. The recording was performed using the SM48 microphone (Shure). The child was guided to hold the microphone at an approximately 10 cm distance from the mouth and to produce the voice in a relaxed state. For the recording and analysis of voice, the Computerized Speech Lab (Model 4150, CSL, Kay Elemetrics, Co) was used. The sample ratio for the voice files was as follows: CSL 11,025 Hz; multidimensional voice program, 50,000 Hz; voice range profile, 44,100 Hz; and real-time pitch, 50,000 Hz.

#### Intervention Environments

Since this study is a home therapy using serious games, treatment was conducted at home in the presence of guardians. Outcome measure was assessed at Jeonbuk National University Hospital by a skilled speech therapist with >3 years of clinical experience.

#### Voice Parameters

##### MPT (seconds) and F0 (Hz)

To measure the MPT using the real-time pitch module, the participants were instructed to vocalize/a/ for the longest possible duration. The F_0_ was measured at the 3-second point from the initial vocalization along the entire stretch of the voice sample.

##### Jitter (%) and RAP (%)

Using the multidimensional voice program module, the participants were instructed to vocalize/a/ for ≥3 seconds three times to obtain the mean of triplicate measurements.

##### Pitch (Hz) and Intensity (dB)

To measure the pitch range using the voice range profile module, the participants were instructed to vocalize/a/ from the lowest range to the highest range he or she could make and, then, vocalize the same sound from the highest range to the lowest range. In addition, the participants were instructed to vocalize/a/ from the highest intensity of voice to the lowest intensity, while the intensity range was measured.

##### VOT

The participants were provided with a set of picture cards corresponding to the word list containing words with a plosive as the initial consonant (/p/, /p^h^/, /p’/, /t/, /t^h^/, /t’/, /k/, /k^h^/, /k’/). Subsequently, they were instructed to look at the card to say or read the word. The word list contained the words used in a study by Gregory and Lee [13] on the development of Korean plosive sounds.

#### Articulation Parameters

##### U-TAP

The U-TAP was used to estimate the articulation accuracy of 43 consonants and 10 vowels at initial, middle, and final positions in words. The word picture cards were used, whereby the participants were instructed to look at the picture card and articulate the word. The sentence-picture cards were used, followed by the articulation of the sentence by the rater, whereby the participants were instructed to repeat after the rater.

##### Three-Position Articulation Test (TA)

The TA was used so that the participants could articulate words containing 2-4 syllables with 19 target consonants at the initial, middle, and final positions in words. The picture cards were presented, and the participants were instructed to articulate the respective words. In analyzing the U-TAP and TA results, the PCC was applied for consonant accuracy using the following equation for PCC calculation:

PCC (%)=the number of correctly articulated consonants/the total number of target consonants×100.

The PCC is widely used as an indicator of the level of articulation disorder because consonants generally reflect a multitude of articulation errors.

##### Alternate Motion Evaluation (DDK)

The DDK rate allows for the evaluation of the oral cavity structure and function and the oral exercise ability. After one or two demonstrations by the rater, the participants were instructed to articulate /pʌ/, /tʌ/, and /kʌ/ to determine the alternating motion rate and /pʌtʌkʌ/ for the sequential motion rate (SMR) within 5 seconds, as rapidly and regularly as possible. The number of syllables articulated per second was estimated.

### Statistical Analysis

Evaluation results at each phase were comparatively analyzed to compare the treatment period with the nontreatment period of the functional game–based home therapy and verify the effects’ retention. All voice data were analyzed using IBM SPSS Statistics (version 24.0; IBM Corp). Repeated-measures ANOVA (RM-ANOVA) was used to compare components of the voice and articulation parameters. The post hoc test was performed as planned with Bonferroni correction at *P*<.05 for variables showing significant values. The significance level was set at .05 in all analyses.

## Results

A total of 13 children (9 males and 4 females) were investigated in this study. The mean age of the participants was 6.94 (SD 2.22) years, with their ages ranging from 4 years to 10 years and 5 months (Table 1).

**Table 1:** Demographic data of the participants.

Participant No.	Age (years)	Sex	U-TAP^a^ (%)	REVT^b^ receptive	REVT expressive
1	7	Male	93.02	106	98
2	6	Male	90.7	68	69
3	8	Male	93.02	97	96
4	7	Male	93.02	82	91
5	6	Male	93.02	73	69
6	4	Male	60.47	44	61
7	4	Male	48.84	59	47
8	7	Female	76.74	82	78
9	4	Male	67.44	61	48
10	10	Female	83.72	130	149
11	4	Male	67.44	54	60
12	4	Female	86.05	64	61
13	4	Female	79.07	36	47

^a^U-TAP: Urimal Test of Articulation and Phonology.

^b^REVT: Receptive and Expressive Vocabulary Test.

### Voice Parameters

**Figure 3:**
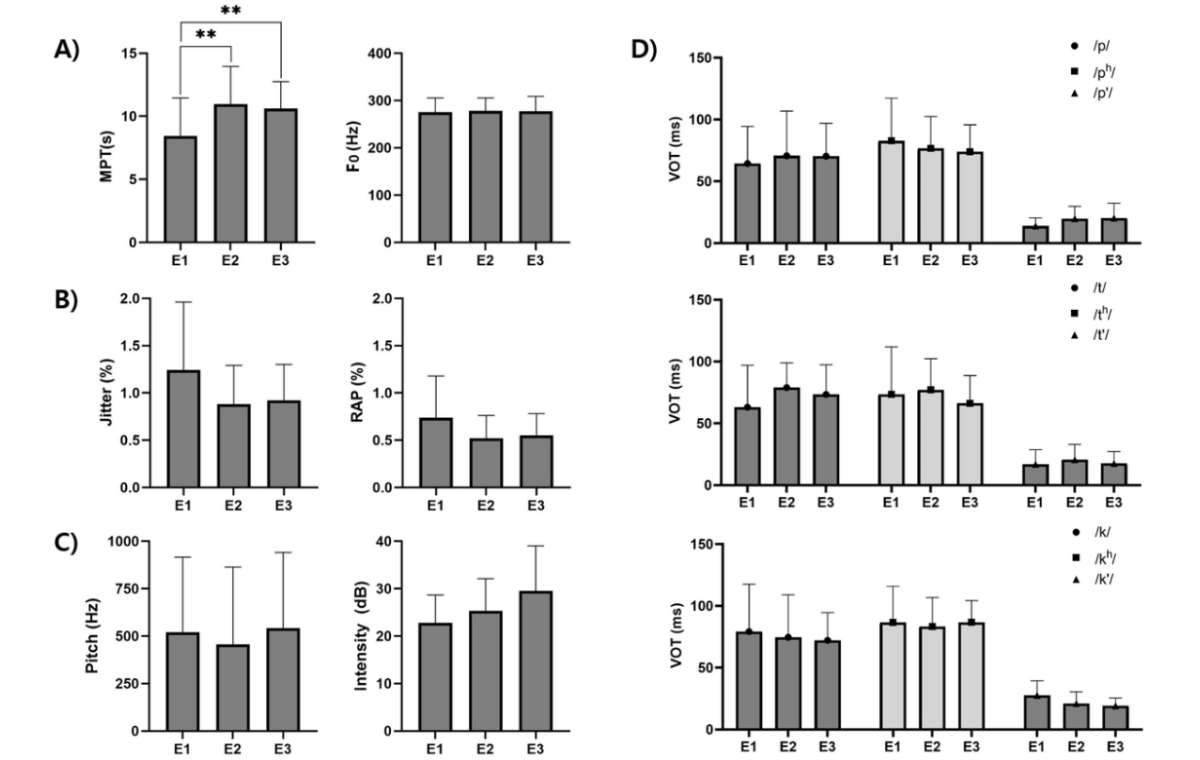
Changes in voice parameters from pre to posttraining. (A) MPT and F0, (B) jitter and RAP, (C) pitch and intensity, and (D) VOT. Data are shown as mean (SD). Repeated-measures ANOVA (RM-ANOVA) with Bonferroni tests was used for statistical analysis. *P<.05, **P<.01. E1: pre-evaluation 1; E2: postevaluation 1; E3: postevaluation 2; F0: frequency; MPT: maximum phonation time; RAP: relative average perturbation; VOT: voice onset time.

A comparison between the treatment and nontreatment periods of functional game–based home therapy indicated a significant effect of the treatment in voice parameters (Figure 3).

### MPT

The MPT showed significant variations across the 3 time points (E1-E2-E3, *P*<.05). In post hoc test, MPT showed differences between phases E1 (mean 8.44, SD 3.02 seconds) and E2 (mean 10.96, SD 2.99 seconds), indicating a significant increase after treatment. In addition, the difference of MPT between phases E1 and E3 (mean 10.62, SD 2.12 seconds) was significant to indicate that the treatment effects had been maintained until 2 months after the training (*P<*.05; Table 2).

**Table 2:** Post hoc analysis of outcome measures.

Variation	Overall Sig Diff^a^	Post hoc^b^		
		△(E1^c^-E2^d^)	△(E1-E3^e^)	△(E2-E3)
MPT^f^	.001	0.007	0.004	1.000
U-TAP^g^ word	<.001	0.003	0.001	1.000
U-TAP sentence	.008	0.037	0.026	1.000
TA^h^	<.001	0.010	0.003	1.000
SMR^i^	.013	0.034	0.061	0.204

^a^Sig Diff: significant difference; analyzed by repeated-measures ANOVA.

^b^Post hoc: Bonferroni test (*P*<.05).

^c^E1: pre-evaluation 1.

^d^E2: postevaluation 1.

^e^E3: postevaluation 2.

^f^MPT: maximum phonation time.

^g^U-TAP: Urimal Test of Articulation and Phonology.

^h^TA: three-position articulation test.

^i^SMR: sequential motion rate.

### Jitter and RAP

The jitter and RAP showed significant variations in RM-ANOVA were noted across the time points (*P*<.05). However, the post hoc test did not indicate significant values for jitter and RAP.

### Other Parameters (F0, Pitch, Intensity, and VOT)

For other materials (F_0_, Pitch, Intensity, and VOT), no significant difference was found between the time points.

### Articulation Parameters

**Figure 4:**
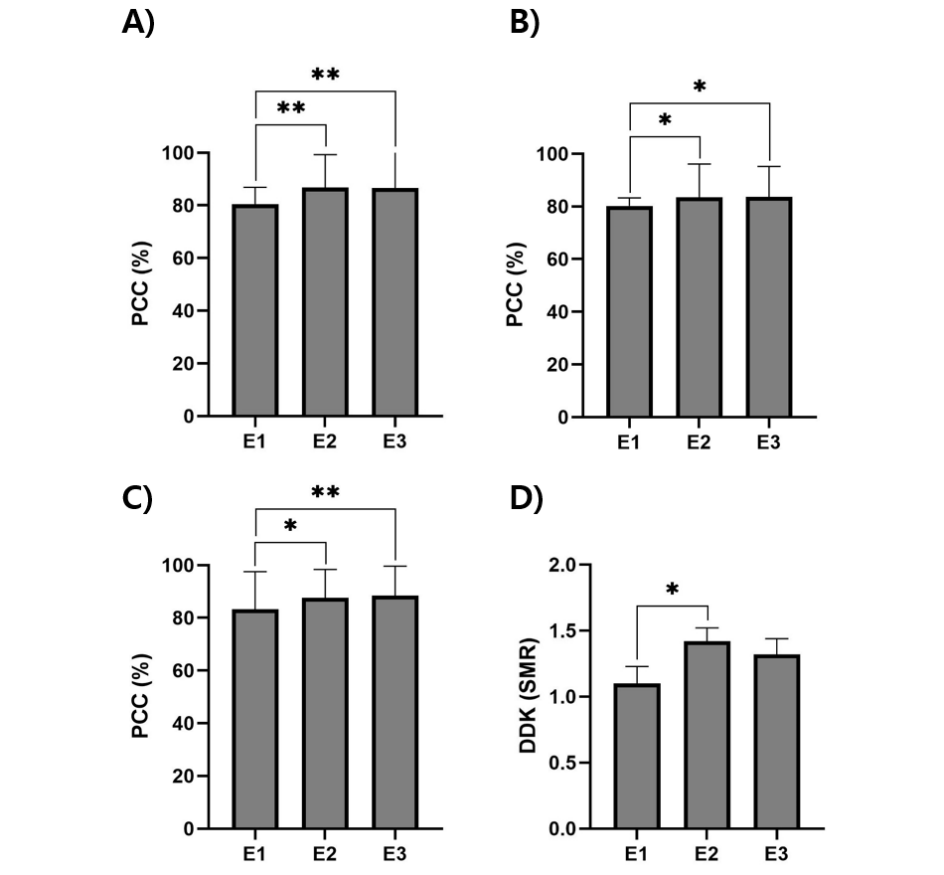
Changes in articulation parameters from pre- to post-training. (A) U-TAP word, (B) U-TAP sentence, (C) TA, and (D) frequency of SMR /pʌtʌkʌ/ of DDK. Data are shown as mean (SD). Repeated-measures ANOVA (RM-ANOVA) with Bonferroni tests was used for statistical analysis. *P<.05, **P<.01. DDK: diadochokinesis; E1: pre-evaluation 1; E2: postevaluation 1; E3: postevaluation 2; PCC: percentage of correct consonants; SMR: sequential motion rate (/pʌtʌkʌ/); TA: three-position articulation test; U-TAP: Urimal Test of Articulation and Phonology.

The functional game–based home therapy achieved a significant effect on articulation parameters (Figure 4).

### U-TAP

The subcategories U-TAP word-unit PCC and sentence-unit PCC showed significant variations in analysis across the 3 time points (*P*<.05). The post hoc test revealed differences between phases E1 (word: mean 80.47, SD 6.38 per sentence; mean 80.23%, SD 3.07%) and E2 (word: mean 86.74, SD 12.55 per sentence; mean 83.49%, SD 12.62%), indicating a significant increase in the U-TAP after treatment. In addition, since there were significant differences between phases E1 and E3 (word: mean 86.51, SD 14.16 per sentence; mean 83.72%, SD 11.50%) and no significant differences between phases E2 and E3, it can be assumed that the treatment effects had been maintained until 2 months after the training (*P*<.05).

### Three-Position Articulation Test (TA)

The subcategories TA PCC showed significant variations in the RM-ANOVA across the time points (*P*<.05). In the post hoc test, TA showed differences between phases E1 (mean 83.18%, SD 14.38%) and E2 (mean 87.50%, SD 10.89%), representing a significant effectiveness after treatment. In addition, as there were significant differences between phases E1 and E3 (mean 88.41%, SD 11.21%) and no significant differences between phases E2 and E3, it can be assumed that representing effectiveness until 2 months after the training (*P*<.05).

### Alternate Motion Evaluation (DDK)

The SMR /pʌtʌkʌ/ of DDK showed significant variations across the time points (*P*<.05). In post hoc test, SMR showed significant differences between phases E1 (mean 1.1, SD 0.14) and E2 (mean 1.42, SD 0.1), indicating improvement of the articulation frequency per second for /pʌtʌkʌ/ after treatment (*P*<.05). However, no significant changes were found between phases E1 and E3 and phases E2 and E3 in the post hoc test.

## Discussion

### Overview

This study conducted a 4-week intervention applying the Smart Speech program consisting of functional games for logopedics to children with articulation disorder. The results of the speech program conducted in this study showed significant changes in both voice and articulation parameters. In voice parameters, MPT, jitter, and pitch showed significant changes in RM-ANOVA. However, post hoc tests showed significant changes between phases E1 and E2 and phases E1 and E3 in the MPT only. Other voice parameters did not show significant differences. In articulation parameters, U-TAP, TA, and DDK showed significant changes in RM-ANOVA. In post hoc tests, U-TAP and TA revealed significant changes between phases E1 and E2 and phases E1 and E3; however, DDK showed significant changes during E1-E2 only.

Several studies have confirmed the effectiveness of conventional treatment methods. However, there is a limitation in that children have to endure time and geographical restrictions for face-to-face treatment [14]. Although the optimum frequency of treatment remains unclear, Williams [15] has suggested that children with articulation disorders may require 30-40 sessions of treatment, depending on the severity and selected treatment method. Long-term intensive training may be effective in children with joint problems; however, economic or geographical constraints may make it difficult to provide treatment adequately [16]. In addition, the stress level in children could increase as the period of intervention increases; hence, a system without time constraints that minimizes training stress in children and allows a guardian’s presence during training should be developed. The importance of home therapy with a parent has been emphasized in the study conducted by Bowen (1998) [17], which further described the available treatment techniques for therapists. The home-based serious game for school-age children enables more flexible scheduling while providing shorter but more frequent intervention sessions, thus, maximizing the usefulness of non–face-to-face treatment.

Computer-assisted logopedics would be advantageous in increasing the treatment frequency even if the treatment is not performed in a direct face-to-face session with a clinician. A computer-assisted home therapy could increase the treatment effects by providing personalized treatment to each patient allowing accessibility for residents living in far regions from the facility or center and, most importantly, providing intensive training and the autonomy that is not possible in conventional programs. Moreover, a familiar and natural environment could ensure psychological stability in children to maximize the treatment effects [18]. Logopedics using a computer program allows for a therapeutic approach at the desired level. As the participants can complete the task the clinician assigned without increasing the time of direct face-to-face treatment, the desired effects can be achieved more rapidly. Moreover, visual representations are provided, and the treatment effects and efficiency of the conventional programs can be improved. Voluntary training is essential in maximizing the treatment effects; however, it may not reach an adequate level because of physical or mental fatigue or lack of volition [19]. In contrast, functional games can motivate children through the added element of fun alongside the advantages of computer-assisted logopedics. Thus, a diversity of functional games for logopedics have been developed to allow the potentially tedious training to be conducted with enhanced focus and fun. The “Vox Games” in Brazil is a logopedics program for children’s vocalization and speech training. “Dr. Speech” in the US offers logopedic-related games based on vocalization, including pitch and intensity. In addition, in Spain, Saz et al [20] have developed a logopedic program for vocalization and articulation training called “Comunica.” In contrast, in Portugal, Grossinho et al [21] have developed “Visual Speech,” a functional game for children with articulation disorder. In the Netherlands, Ganzeboom et al [22] had recently applied the “Treasure Hunters,” a functional game of logopedics, in 5 patients with Parkinson disease or stroke and reported its positive effects on speech intelligibility. Hair et al [23] have developed the “Apraxia World,” a game of logopedics for children with difficulty in speech production, and investigated the preference and satisfaction with using the game in 14 children with articulation disorder (4-12 years). Therefore, various speech therapy games have been developed based on each country’s language, and “Smart Speech” is a serious game based on the Korean language.

Recently, various functional games have been applied in logopedics, whereas numerous computer-assisted logopedics for articulation disorder have been developed and tested; however, there is a comparative lack of studies investigating the clinical effects. Thus, this study applied a functional game–based home therapy to children with articulation disorder and, through preintervention and postintervention tests of voice, articulation, and alternate motion, investigated whether the therapy had positive effects on phonation ability and whether positive changes were maintained. In voice parameters, only MPT showed significant changes during E1-E2 and E1-E3 in post hoc study. Other voice parameters did not show significant changes. The ability to maintain vocalization is associated with the expiratory pressure in the respiratory organs. The MPT is a task of speech-motor evaluation that can predict the level of coordination among the muscles related to respiration, vocalization, articulation, and resonance [24]. Thus, the MPT results in this study indicated an increased ability of coordination in breathing and vocalization mechanisms by breathing and vocalization training through functional games. Breathing training of “Smart Speech” focused on expiration, and therefore, it positively affected the increase in phonation time. The acoustic analysis as voice parameters could be useful for identifying improvement in patients with cerebral palsy who showed dysphonia or dysarthria. For the articulation parameters, U-TAP and TA showed significant changes during E1-E2 and E1-E3, whereas DDK showed significant changes during E1-E2 only. It suggests that the effects of oral exercise and articulation training increased the PCC and DDK. In the “Smart Speech” program, oral exercise and speech training had many components that may have improved articulation rather than vocalization. It can be assumed that these factors made the following results. This coincided with the findings of Ahn [25], who reported the positive effect of oral exercise training on consonant accuracy, and with those of Lee et al [26], who applied an AR game–based word and sentence training to improve the word and sentence articulation accuracy in children with articulation disorder. Further, the results agreed with the findings of Lohman-Hawk [27], who reported that the DDK rate in patients with articulation disorder increased by 50% through treatment involving oral exercise training. Our findings suggested that functional game–based home therapy could prove useful in planning logopedic interventions in clinical practice. When children used Smart Speech programs at home, the guardians ensured proper treatment compliance. A clinical researcher contacted the guardian by telephone once a week to monitor the home-based therapy. It should be noted, however, that patients and guardians would be faced with difficulties in planning and conducting suitable training, and the participation and counseling of clinicians will be required.

### Principal Results

In this study, children showed significant therapeutic effects in most articulatory indicators but did not show significant therapeutic effects other than MPT in negative indicators. A previous study on the VOT of children with functional articulation disorders has reported that the change in VOT according to the articulation position of plosives and vocalization type of children with functional articulation disorders was the same as that of ordinary children and that speech motor control skill was normally developed in the production of the plosives [28]. This suggests that speech problems in children with functional articulation disorders are more likely to occur owing to the decline of motor control of other articulation structures, compensation for speech motor control, and speech programming skills than in the upper laryngeal structure. The F_0_ representing the frequency of vocal cords per second, the jitter representing vibration speed, and the shimmer representing vibration size are directly related to the anatomical structure of the larynx and can be affected by age or sex. This is more related to anatomical factors, and it is difficult to expect a significant therapeutic effect through training because it is less likely that children’s articulation disorders will occur due to dysregulation of the upper and laryngeal structures [29]. The results of this study showed a correlation with previous studies and confirmed that the speech motor control or programming ability of articulation structures is likely to be sufficiently improved through a systematic home-based serious game. As the children who participated in this study did not have anatomical problems, it is highly possible that there was no problem with the tremor of the vocal cords. Except for 2 children, most participants showed initial evaluation for F_0_ and jitter within the normal range. Thus, in this study, voice parameters may have shown no significant changes. Additionally, in the composition of the “Smart Speech” program, the treatment time of oral exercise and speech training was relatively longer than that of the breaking training, which would have affected the improvement of the articulation parameter.

### Limitations

Our study had several limitations. First, the sample size was relatively small. The small sample size may have led to unstable results. Follow-up studies need to analyze a wider population. Second, this study was a single-arm, nonrandomized study. This study was designed as a randomized controlled trial, but it was difficult to set up sham groups because all guardians wanted treatment, and it was difficult to set up other home-based treatments to apply to control groups other than serious games. Therefore, the period of maintaining daily life without treatment was set as a control, and a single-arm design was adopted to assess and compare the period of home-based therapy as an experimental period. As there was no control group, follow-up studies, including the control group, are required to accurately verify the treatment effect. Third, the same treatment was performed regardless of the patient’s baseline score, and there is a limitation in that selective treatment according to the patient’s condition was not provided. Fourth, Children with a substantially wide range of age and severity were included in the study. In further studies, we need to narrow the scope of participants’ age or seriousness of speech sound disorders. Fifth, In this study, we tried to analyze various indicators, but not all articulation parameters were measured due to limited time and human resources. In subsequent studies, we will perform as many standard assessments as possible and analyze the therapeutic effect in more detail by comparing initial and end time points. Finally, the follow-up period in this study was relatively short. Thus, further research is warranted to establish the long-term effectiveness of home-based serious games.

### Conclusions

In conclusion, after applying a home-based serious game (Smart Speech) to children with articulation disorders, voice parameters showed improvement in MPT, and articulation parameters revealed improvement in all U-TAP, TA, and DDK. Therefore, home-based serious games are considered to be helpful in improving language function.

## Appendix

Multimedia Appendix 1 CONSORT-eHEALTH Checklist (V 1.6.1).

## Abbreviations

DDK: diadochokinesis
F0: frequency
MPT: maximum phonation time
PCC: percentage of correct consonants
RAP: relative average perturbation
RM-ANOVA: repeated-measures ANOVA
SMR: sequential motion rate
TA: three-position articulation test
U-TAP: Urimal Test of Articulation and Phonology
VOT: voice onset time
